# Myelodysplastic/myeloproliferative neoplasms with ring sideroblasts and thrombocytosis (MDS/MPN-RS-T): Mayo-Moffitt collaborative study of 158 patients

**DOI:** 10.1038/s41408-022-00622-8

**Published:** 2022-02-01

**Authors:** Abhishek A. Mangaonkar, Terra L. Lasho, Rhett P. Ketterling, Kaaren K. Reichard, Naseema Gangat, Aref Al-Kali, Kebede H. Begna, Animesh Pardanani, Najla H. Al Ali, Chetasi Talati, David Sallman, Eric Padron, Mrinal M. Patnaik, Ayalew Tefferi, Rami Komrokji

**Affiliations:** 1 Division of Hematology, Department of Medicine, Mayo Clinic, Rochester, MN USA; 2 Department of Laboratory Medicine and Pathology, Mayo Clinic, Rochester, MN USA; 3 Department of Malignant Hematology, H. Lee Moffitt Cancer Center, Tampa, FL USA

**Keywords:** Myelodysplastic syndrome, Cancer genomics

## Abstract

The current World Health Organization (WHO) classification of myeloid malignancies includes myelodysplastic/myeloproliferative neoplasms with ring sideroblasts and thrombocytosis (MDS/MPN-RS-T) as a distinct entity. Previous literature on predictors of survival was based on the provisional category of refractory anemia with ring sideroblast and thrombocytosis (RARS-T), which was not subject to MDS/MPN-RS-T exclusionary criteria such as PB blast% ≥1, BM blast% ≥5 or cytogenetic abnormalities such as t(3;3)(q21.2;q26.2), inv(3)(q21.23q26.2) or isolated del(5q). We examined overall (OS) and leukemia-free (LFS) survival and its predictors, among 158 patients with WHO-defined MDS/MPN-RS-T. In univariate analysis, age ≥70 years (*P* = 0.006), hemoglobin (Hb) ≤10 g/dL (*P* = 0.03) and abnormal karyotype (excluding -Y, *P* = 0.008) were associated with shortened OS, which was otherwise not affected by either *ASXL1* (*P* = 0.7), *SF3B1 (P* = 0.4*)* or *JAK2* V617F *(P* = 0.7*)* mutations; in multivariable analysis, Hb ≤ 10 g/dL (*P* = 0.03) and abnormal karyotype (*P* = 0.001) remained significant, and thus allowed the development of an operational survival model with low (0 risk factors, median OS 10.5 years), intermediate (1 risk factor, median OS 4.8 years) and high risk (2 risk factors, median OS 1.4 years) categories (*P* = 0.0009). Comparison of MDS/MPN-RS-T (*n* = 158) and MDS/MPN-U with BM RS ≥ 15% (MDS/MPN-U-RS; *n* = 25) did not reveal significant differences in frequency of thrombosis, OS, or LFS, although *SF3B1* mutation frequency was higher in the former (93% versus 59%; *P* = 0.0005). These data suggest limited survival impact for molecular abnormalities and the morphological distinction between MDS/MPN-RS-T and MDS/MPN-U-RS.

## Introduction

The 2016 World Health Organization (WHO) revision of myeloid malignancies and its recent iteration included patients with myelodysplastic/myeloproliferative neoplasms with ring sideroblasts and thrombocytosis (MDS/MPN-RS-T) as a distinct entity, under the MDS/MPN category [1, 2]. Previously, these patients were included under the provisional MDS/MPN category of refractory anemia with ring sideroblasts and thrombocytosis (RARS-T) [3, 4]. MDS/MPN-RS-T is distinguished from myelodysplastic syndrome with ring sideroblasts (MDS-RS) by the presence of thrombocytosis (platelet count ≥450 × 10^9^/L) and bone marrow (BM) megakaryocyte morphology similar to that seen in MPN. MDS/MPN-RS-T is in addition characterized by presence of *SF3B1* mutations in 60–90% of patients and *JAK2 V617F* in a smaller percentage [1, 5–10]. In order to be classified under the MDS/MPN-RS-T category, patients should also meet certain exclusionary criteria such as BM blast% ≥5, peripheral blood (PB) blast% ≥1, and cytogenetic abnormalities such as t(3; 3)(q21.3; q26.2), inv(3)(q21.3; q26.2) or isolated del(5q), or the *BCR/ABL1* fusion oncogene [1, 11]. Of note, suspected cases with ≥15% BM RS and ≥1% PB or ≥5% BM blasts are classified as MDS/MPN-unclassifiable (MDS/MPN-U), while cases with leukocytosis [white blood cell (WBC) count of ≥13 × 10^9^/L] might be included in the category of MDS/MPN-RS-T, even in the absence of thrombocytosis.

We have previously reported data from a large cohort of patients (*n* = 135) with MDS/MPN-U and found that patients with BM RS ≥ 15% (*n* = 13) had better outcomes than those with BM RS < 15% (*n* = 103), but similar outcomes when compared to patients with MDS/MPN-RS-T patients (*n* = 72) [12]. The analysis was however limited by small sample size and differential follow-up between the two groups. Nonetheless, these data call into question the necessity of a strict MDS/MPN-RS-T definition based on the aforementioned criteria and highlight its limitations. The objectives of the current study were to (i) define the natural history of MDS/MPN-RS-T, including determination of long-term survival and prognostic factors, and (ii) formally compare clinical and prognostic features between a larger cohort of patients with MDS/MPN-RS-T (*n* = 158) and MDS/MPN-U with ≥15% BM RS (MDS/MPN-U-RS), but not meeting criteria for MDS/MPN-RS-T (*n* = 25), so as to inform future revisions of the WHO classification of myeloid malignancies.

## Methods

Clinical and pathologic data from consecutive adult patients with WHO-defined MDS/MPN-RS-T (*n* = 158) and MDS/MPN-U-RS (*n* = 25) were collected from two major institutions, Mayo Clinic (Rochester, Minnesota) and H. Lee Moffitt Cancer Center (Tampa, Florida) after institutional review board (IRB) approval at each institution. Diagnostic BM biopsy reports were carefully reviewed to ensure compliance with the latest WHO criteria. The MDS/MPN-U-RS patients did not meet MDS/MPN-RS-T criteria due to PB blast% >1 (*n* = 9), BM blast% >5 (*n* = 8), and the rest due to absence of thrombocytosis. There were 3 MDS/MPN-U-RS patients who did not have thrombocytosis or leukocytosis but were classified as such since they did not meet diagnostic requirements of any other MDS/MPN category. Among these three patients, two had predominance of MPN features on bone marrow assessment (atypical megakaryocytes, and/or mild reticulin fibrosis) and one had BM blast% >5. Next generation sequencing (NGS) was done at diagnosis or first referral using institutional or commercially available myeloid malignancy-specific gene panels (details in Supplementary Table 1). Distribution of continuous variables was statistically compared using nonparametric (Mann–Whitney or Kruskal–Wallis) tests, while nominal variables were compared using the Chi-Square test. Time to event analyses used the method of Kaplan–Meier for univariate comparisons. Significant (*P* < 0.05) continuous variables were converted into categorical variables after derivation of cut-off points through a receiver operating curve (ROC) analysis and were chosen for multivariate analysis performed through the Cox proportional hazard regression model. Overall survival (OS) was computed from the date of diagnosis to date of death or last follow-up. Leukemia-free survival (LFS) was calculated from date of diagnosis to date of acute myeloid leukemia (AML) transformation or last follow-up; in other words, AML transformation replaced death as the uncensored variable in calculating LFS. Patients who underwent allogeneic hematopoietic stem cell transplantation were censored at the time of transplantation.

## Results

Overall, 183 patients were included in the study: 158 MDS/MPN-RS-T and 25 MDS/MPN-U-RS. The median age of the MDS/MPN-RS-T cohort was 71 (range: 38–94) years; 83 (52%) males. At last median follow-up of 62 [95% confidence interval (CI) 45–80) months, there were 66 (42%) deaths and 6 (4%) AML transformations. The Kaplan–Meier estimates of median OS and LFS were 6 (95% CI 5–9) and 3 (95% CI 2–4) years, respectively. Cytogenetic abnormalities (excluding -Y) were present in 11 (15%) patients with the most frequent ones being +8 (*n* = 4, 36%), 20q- (*n* = 2, 18%), and 7q- (*n* = 2, 18%), complex (*n* = 2, 18%) and monosomal karyotype (*n* = 2, 18%, Table 1). International Prognostic Scoring System (IPSS) cytogenetic stratification included 63 (88%) good, 4 (6%) intermediate and 5 (7%) poor risk categories, while the revised IPSS (IPSS-R) cytogenetic stratification included 5 (7%) very good, 59 (81%) good, 4 (5%) intermediate, 3 (4%) poor and 2 (3%) very poor risk categories, respectively. Frequent molecular abnormalities included *SF3B1* [*n* = 103, 93%, most commonly K700E 52%, H662Q 12%, and K666R 7%], *JAK2 V617F* (*n* = 13, 30%), *ASXL1* (*n* = 11, 22%), *DNMT3A* (*n* = 6, 14%), *SETBP1* (*n* = 4, 10%), and *TET2* (*n* = 3, 7%) among others (Table 1). Information on therapy was available for MDS/MPN-RS-T patients in the Mayo cohort (*n* = 78); 44 (56%) were treated with erythropoietin stimulating agents, 16 (21%) lenalidomide, 4 (5%) investigational agents, while 1 each was treated with danazol, hydroyxurea, and splenectomy, respectively.

**Table 1:** Table showing phenotypic and genomic differences among patients with WHO-defined MDS/MPN-RS-T and MDS/MPN-U patients with BM RS% ≥ 15 (MDS/MPN-U-RS).

Variable; Median value (range or %)	MDS/MPN-RS-T (*n* = 158)	MDS/MPN-U-RS (*n* = 25)	*P-value*
Age (years)	71 (38–94)	71 (37–86)	0.9
Age ≥70 (years)	96 (61)	13 (52)	0.4
Males (%)	82 (52)	9 (36)	0.1
Clinical parameters	*Evaluable* = *78*	*Evaluable* = *25*	
Hb; g/dL	9.5 (6.6–14.5)	9 (5.6–12.7)	0.2
Hb ≤10 g/dL	51 (65)	18 (72)	0.5
WBC count × 10^3^ per µL	7.6 (1.9–25.8)	6.9 (1.7–48.9)	0.5
Platelet count × 10^3^ per µL	585 (454–1741)	559 (14–1798)	0.7
PB blast%	–	0 (0–9)	<0.0001^a^
PB blasts ≥1%	–	9	<0.0001^a^
BM blast%	1 (0–4)	3 (0–8)	0.001^a^
BM blast ≥5%	–	8	<0.0001^a^
BM RS%	47.5 (15–90)	35 (15–80)	0.2
Thrombosis %	14 (18)	2 (8)	0.3
Arterial	6 (43)	1 (50)	
Venous	6 (43)	1 (50)	
Cytogenetics	*Evaluable* = *72*	*Evaluable* = *24*	
Abnormal karyotype (except-Y)	11 (15)	4 (17)	0.9
Monosomal karyotype	2 (3)	2 (8)	0.3
Complex karyotype	2 (3)	2 (8)	0.3
IPSS cytogenetics			
Good	63 (88)	20 (83)	0.7
Intermediate	4 (6)	1 (4)	
Poor	5 (7)	3 (13)	
IPSS-R cytogenetics			
Very good	5 (7)	–	0.4
Good	59 (81)	20 (83)	
Intermediate	4 (5)	1 (4)	
Poor	3 (4)	1 (4)	
Very poor	2 (3)	2 (8)	
Pathogenic variants			
* ASXL1*	Evaluable = 49; 10 (20)	Evaluable = 17; 4 (24)	0.9
* DNMT3A*	Evaluable = 43; 6 (14)	Evaluable = 15; 2 (13)	0.9
* TET2*	Evaluable = 43; 3 (7)	Evaluable = 15; 2 (13)	0.5
* SF3B1*	Evaluable = 111; 103 (93)	Evaluable = 17; 10 (59)	0.0005^a^
* SRSF2*	Evaluable = 43; 1 (2)	Evaluable = 15; 2 (13)	0.1
* ZRSR2*	Evaluable = 43; 1 (2)	Evaluable = 15; 0 (0)	0.4
* U2AF1*	Evaluable = 43; 1 (2)	Evaluable = 15; 2 (13)	0.1
* JAK2 V617F*	Evaluable = 43; 13 (30)	Evaluable = 15; 2 (13)	0.2
* CALR*	Evaluable = 43; 2 (5)	Evaluable = 15; 0 (0)	0.3
* SETBP1*	Evaluable = 40; 4 (10)	Evaluable = 15; 2 (13)	0.7
* TP53*	Evaluable = 39; 1 (2)	Evaluable = 15; 0 (0)	0.4
Outcomes (evaluable = 183)			
Median follow-up (months)	62 (45–80)	53 (18–123)	0.9
Leukemic transformation (%)	6 (4)	–	0.0003^a^
LFS (95% CI) years	Median not reached	Median not reached	0.4
OS (95% CI) years	6 (5–9)	7.3 (1.8–11.4)	0.3

*MDS/MPN-RS-T* myelodysplastic/myeloproliferative neoplasms with RS and thrombocytosis, *MDS/MPN-U* myelodysplastic/myeloproliferative neoplasms, unclassifiable, *Hb* hemoglobin, *BM* bone marrow, *RS* ring sideroblast, *IPSS* International Prognostic Scoring System, *IPSS-R* Revised International Prognostic Scoring System, *LFS* acute myeloid leukemia-free survival, *OS* overall survival.

^a^Denotes statistical significance (*P* < 0.05).

**Fig. 1:**
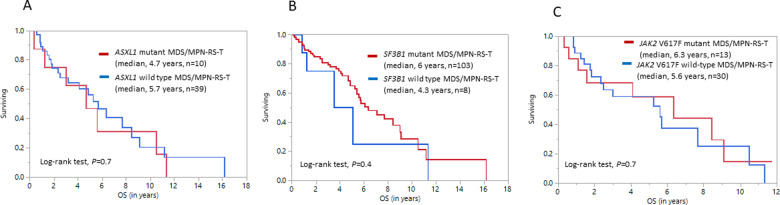
Figure showing overall survival (OS) differences between *ASXL1*, *SF3B1* and *JAK2* V617F mutated and wild-type MDS/MPN-RS-T patients. **A** shows that there is no significant Kaplan–Meier estimate of OS difference between *ASXL1* mutated and wild-type MDS/MPN-RS-T patients [median OS, 4.7 (95% CI 0.3–10.5) versus 5.7 (95% CI 2.5–8.4) years, *P* = 0.7], **B** shows that there is no significant Kaplan–Meier estimate of OS difference between *SF3B1* mutated and wild-type MDS/MPN-RS-T patients [median OS, 6.3 (95% CI 5.4–9) versus 4.3 (95% CI 0.8–11.3) years, *P* = 0.4], **C** shows that there is no significant Kaplan–Meier estimate of OS difference between *JAK2* V617F mutated and wild-type MDS/MPN-RS-T patients [median OS, 6.3 (95% CI 1.1–9.1) versus 5.6 (95% CI 2.3–10.5) years, *P* = 0.7].

**Fig. 2:**
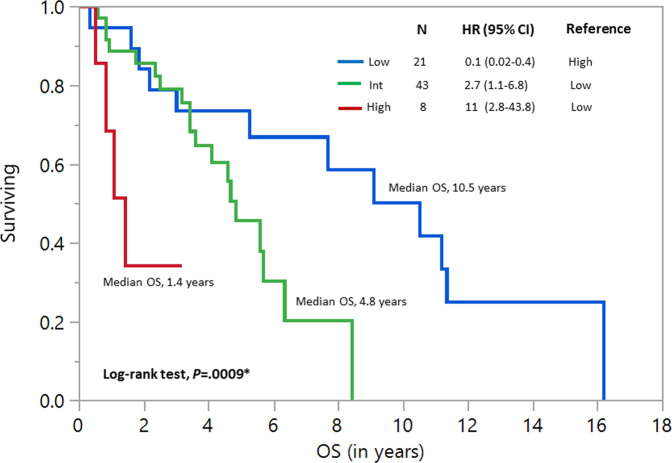
Figure showing operational survival model using hemoglobin ≤ 10 gm/dL and abnormal karyotype (excluding -Y) identified as independent significant variables using multivariate analysis. Patients were diving into three survival categories; low [median OS 10.5 years 95% CI (3–11.3)], intermediate [median OS 4.8 years 95% CI (3.4–6.3) and high-risk [median OS 1.4 years, 95% CI (0.5-not reached), *P* = 0.0009] categories. Statistical significance (*P* < 0.05) is denoted by *, calculated through the log-rank test.

In univariate analysis, age ≥70 years (HR 2, 95% CI 1.2–3.7, *P* = 0.005), hemoglobin ≤10 g/dL (HR 2.4, 95% CI 1.1–5.3, *P* = 0.03), and abnormal karyotype (HR 3.7, 95% CI 1.4–9.5, *P* = 0.008) independently predicted shorter survival. In multivariate analysis, hemoglobin ≤10 g/dL (HR 3.12, 95% CI 1.1–8.5, *P* = 0.03) and abnormal karyotype (HR 7.3, 95% CI 1.1–8.5, *P* = 0.001) retained prognostic significance (Table 2). Importantly, none of the molecular abnormalities (Fig. 1), including *ASXL1* (*P* = 0.7, Fig. 1A), *SF3B1 (P* = 0.4, Fig. 1B) *or JAK2* V617F *(P* = 0.7, Fig. 1C), platelet count (*P* = 0.2) or BM blast% (*P* = 0.6) predicted survival. *SF3B1* VAF information was available in only 43 patients, and was also not prognostically relevant (*P* = 0.3). However, when only frameshift and nonsense *ASXL1* mutations were considered, they were able to predict an adverse OS (*P* = 0.02) in a univariate analysis, but not in a multivariate model (*P* = 0.1). The prognostic irrelevance of molecular abnormalities such as *ASXL1 (P* = 0.9*)*, *SF3B1 (P* = 0.4*)*, or *JAK2 V617F (P* = 0.8) persisted even in MDS/MPN-RS-T patients with a normal karyotype (*n* = 61). An operational survival model using hemoglobin ≤10 g/dL (1 point) and abnormal karyotype (1 point) delineated patients into low [0 points, median OS 10.5 years, 95% CI (3–11.3)], intermediate [1 point, median OS 4.8 years, 95% CI (3.4–6.3)] and high-risk (2 points, median OS 1.4 years, 95% CI (0.5-not reached)] survival categories (Fig. 2). Among the high-risk patients (*n* = 8), three carried chromosome 7 abnormalities (1 with monosomy 7), two had a complex monosomal karyotype and 1 patient each had *ASXL1* and *TP53* mutations at diagnosis, respectively.

**Table 2:** Univariate and multivariate analysis of clinical and genomic characteristics among patients with MDS/MPN-RS-T.

Variable	Univariate analysis *P-*value (HR, 95% CI)	Multivariate analysis *P-*value (HR, 95% CI)
Age (years)	<0.0001 (1.1, 1–1.1)	–
Age ≥70 (years)	0.006 (2, 1.2–3.7)^a^	0.8 (1.1, 0.4–3)
Male gender	0.2 (1.4, 0.9–2.3)	–
Hemoglobin (g/dL)	0.004 (1.4, 0.5–1)^a^	–
Hemoglobin ≤10 (g/dL)	0.03 (2.4, 1.1–5.3)^a^	0.03 (3.12, 1.1–8.5)^a^
WBC count (x10^3^/µL)	0.5 (1, 0.9–1)	–
Platelet count (x10^3^/µL)	0.2 (1, 0.99–1)	–
BM blast%	0.6 (1.1, 0.7–1.7)	–
BM RS%	0.6 (1, 0.97–1.01)	–
Bone marrow fibrosis (any grade)	0.1 (2.4, 0.8–7.2)	–
Abnormal karyotype (excluding -Y)	0.008 (3.7, 1.4–9.5)^a^	0.001 (7.3, 2.2–23.8)^a^
IPSS cytogenetic categories		
Good	Reference	–
Intermediate	0.04 (3.7, 1.1–12.6)^a^	–
Poor	0.1 (3.5, 0.8–15.8)	–
IPSS-R cytogenetic categories		
Very good	Reference	–
Good	0.07 (0.4, 0.1–1.1)	–
Intermediate	0.9 (0.9, 0.2–5)	–
Poor	0.1 (4.2, 0.7–24.6)	–
Very poor	0.6 (1.9, 0.2–18.6)	–
Molecular abnormalities (only included if *n* ≥ 3)		
* ASXL1*	0.7 (1.2, 0.5–2.9)	–
* ASXL1 (only frameshift and nonsense)*	0.02 (3.3, 1.2–9)^a^	0.1 (2.9, 0.8–10.1)
* TET2*	0.1 (1.8, 0-NR)	–
* DNMT3A*	0.3 (0.3, 0.04–2.3)	
* SF3B1*	0.4 (0.6, 0.2–1.7)	–
* JAK2*	0.7 (0.9, 0.4–2.01)	–
* SETBP1*	0.75 (1.2, 0.4–3.7)	–

Significant variables (continuous variables were converted into categorical variables after derivation of cut-off points through a ROC analysis) in a univariate analysis were chosen for multivariate analysis, which was performed through Cox proportional hazard model.

*MDS/MPN-U* myelodysplastic/myeloproliferative neoplasms with ring sideroblasts and thrombocytosis, *ROC* receiver operating characteristic, *HR* hazard ratio, *WBC* white blood cell, *BM* bone marrow, *RS* ring sideroblast; *IPSS* International Prognostic Scoring System, *IPSS-R* Revised International Prognostic Scoring System, *NR* not reached.

^a^Denotes statistical significance, *P* < 0.05) in predicting overall survival.

**Fig. 3:**
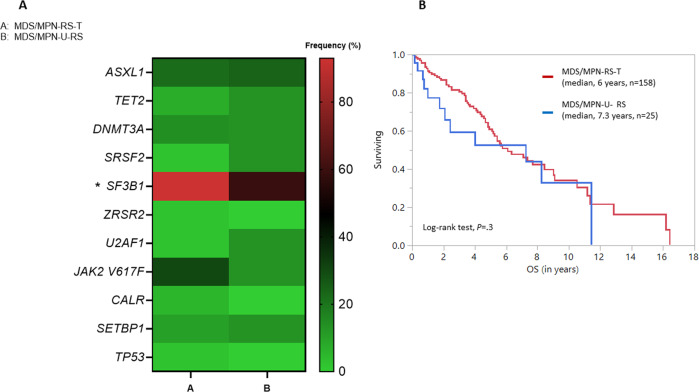
Figure showing molecular landscape, OS and LFS differences between WHO-defined MDS/MPN-RS-T and MDS/MPN-U patients with BM RS ≥ 15% (MDS/MPN-U-RS). **A** shows a heatmap displaying differences in mutational frequencies between MDS/MPN-RS-T and MDS/MPN-U-RS. *SF3B1* is the only gene mutated at a significantly (*) higher frequency in the MDS/MPN-RS-T group. **B** shows that the Kaplan–Meier estimate of median OS (95% CI) did not differ significantly between the two groups [6 (5–9) versus 7.3 (1.8–11.4) years, *P* = 0.3]. Log-rank test was used to calculate the *P*-value.

Comparison of the 158 patients with MDS/MPN-RS-T and the 25 patients with MDS/MPN-U-RS revealed a higher frequency of *SF3B1* mutations (92% versus 40%, *P* = 0.003, Fig. 3A) and AML transformation rate (4% versus 0%, *P* = 0.0003) in MDS/MPN-RS-T (Table 1). Overall (median, 6 versus 7.3 years, *P* = 0.3, Fig. 3B) and leukemia-free (median not reached in either groups, *P* = 0.4) survival between the two groups was similar. Frequency of thrombosis (overall *n* = 14, 7-arterial, 7-venous, 11-before diagnosis, 3-after diagnosis) was also similar in the two groups (18% versus 8%, *P* = 0.3, Table 1). Combined analysis of both groups did not identify platelet count <450 × 10^9^/L (HR 1.1, 95% CI 0.3–4.7, *P* = 0.9), BM blast% ≥5 (HR 1.2 95% CI 0.3–4.96, *P* = 0.8) or PB blast% ≥1 (HR 2.4, 95% CI 0.94–6.2, *P* = 0.07) to be prognostically relevant for OS (could not assess for LFS due to limited events). Similar to MDS/MPN-RS-T, age ≥70 years (HR 2.04, 95% CI 1.3–3.3, *P* = 0.004), Hb ≤10 g/dL (HR 2.2, 95% CI 1.14–4.1, *P* = 0.02), and abnormal cytogenetics [except -Y, HR 2.4, 95% CI 1.12–5.4, *P* = 0.02] significantly predicted inferior OS. Additionally, in the MDS/MPN-U-RS group (*n* = 25), platelet count (*P* = 0.9), BM blast% (*P* = 0.8) or PB blast% (*P* = 0.2) remained prognostically irrelevant for OS prediction.

## Discussion

MDS/MPN-RS-T patients, previously classified under the provisional WHO category of RARS-T, have been reported to have a median OS around 6 years, and leukemic transformation rate of ~2% [13–15]. Previously, hemoglobin <10 gm/dL, abnormal karyotype, *ASXL1*, and *SETBP1* mutations have been found to significantly predict adverse OS [13]; however, these data were based on RARS-T patients, which were not subject to MDS/MPN-RS-T exclusionary criteria such as PB blast% ≥1, BM blast% ≥5 or cytogenetic abnormalities such as t(3;3)(q21.2;q26.2), inv(3)(q21.23q26.2) or isolated del(5q). Further, *ASXL1* and *SETBP1* mutations were not statistically significant in a multivariate model [13]. When we reclassified our patients as per the contemporary WHO definition of MDS/MPN-RS-T, only Hb ≤10 gm/dL and abnormal karyotype (without -Y) retained independent prognostic significance. Although the presence of any *ASXL1* mutation was unable to predict an adverse OS in a univariate analysis; frameshift and nonsense *ASXL1* mutations were able to do so, similar to previously published studies [13, 16]. Another study identified *SF3B1* and *JAK2V617F* as independent predictors of favorable prognosis, however these could not be validated in our cohort [17]. These data highlight that the contemporary WHO-defined MDS/MPN-RS-T category is clinically and molecularly less heterogenous than RARS-T, and yet this classification has key limitations. An operational survival model relying on hemoglobin ≤10 g/dL (1 point) and abnormal karyotype (1 point) was able to risk stratify MDS/MPN-RS-T patients into low (0 points), intermediate (1 point), and high-risk categories (2 points), respectively. High-risk MDS/MPN-RS-T patients have an inferior median OS (1.4 years) and can be considered for aggressive therapeutic strategies such as an allogeneic hematopoietic stem cell transplantation. However, such a risk-adapted management approach would need prospective validation.

The contemporary MDS/MPN-RS-T definition excludes patients with MDS/MPN-U with BM RS ≥ 15% if the platelet count is less than 450 × 10^9^/L, PB blast ≥1% or BM blast% ≥5. In our study, when we combined MDS/MPN-RS-T patients with MDS/MPN-U-RS, the platelet count, PB blast% or BM blast% did not predict for OS or LFS. Relevant clinical parameters such as frequency of thrombosis also did not significantly differ between the two groups. The prognostic irrelevance of platelet count, PB blast% or BM blast% remained true when MDS/MPN-U-RS cohort was separately analyzed. Application of these parameters as exclusion criteria for MDS/MPN-RS-T is therefore too restrictive and leaves out certain patients with MDS/MPN-U-RS, who have identical outcomes to MDS/MPN-RS-T patients as per the current definition. Limitations of the study include retrospective nature, heterogenous clinical and genomic information in the two institutional cohorts, and limited sample size.

In conclusion, the findings from the current study did not identify molecular abnormalities as predictors of survival in MDS/MPN-RS-T, and also argue against the use of currently applied exclusionary criteria in defining MDS/MPN-RS-T, separate from MDS/MPN-U-RS. In other words, our observations support unification of MDS/MPN-U-RS and MDS/MPN-RS-T under a new category of “MDS/MPN-RS”, pending independent external validation. This will allow uniform inclusion of MDS/MPN patients with RS in future clinical trials with novel drugs focused on reversing erythroid arrest such as TGFβ modulators, splicing inhibitors among others [18–20].

## Supplementary information

Supplementary table 1S
