# Supplementary material for: Myelodysplastic/myeloproliferative neoplasms with ring sideroblasts and thrombocytosis (MDS/MPN-RS-T): Mayo-Moffitt collaborative study of 158 patients
Source: Blood Cancer J. 2022 Feb 1;12(2):26. doi: 10.1038/s41408-022-00622-8 (PMC8807827; doi:10.1038/s41408-022-00622-8)
Supplement: Supplementary file 1 — Supplementary table 1S [file 41408_2022_622_MOESM1_ESM.docx]

***Table 1S:*** Table showing the details of various next generation sequencing (NGS) panels used for testing patients included in the study.

| Gene panel | Institution | List of genes | Coding region coverage | Read depth |
| --- | --- | --- | --- | --- |
| OncoHeme NGS for Hematologic Cancers | Mayo Clinic | *ASXL1; BCOR; BRAF; CALR; CBL; CEBPA; CSF3R; DNMT3A; ETV6; EZH2; FLT3; GATA1; GATA2; IDH1; IDH2; JAK2; KIT; KRAS; MPL; MYD88; NOTCH1; NPM1; NRAS; PHF6; PTPN11; RUNX1; SETBP1; SF3B1; SRSF2; TERT; TET2; TP53; U2AF1; WT1; ZRSR2* | Variable per gene: see details at https://www.mayomedicallaboratories.com/test-catalog/Overview/63367 | >250X |
| Research NGS | Mayo Clinic | *ASXL1; ASXL2; ATM; BCOR; BCORL1; CALR; CBL; CEBPA; CSF3R; DNMT3A; EED; ETNK1; EZH2; FLT3; GATA2; IDH1; IDH2; JAK2; JARID2; KIT; KRAS; MPL; NRAS; PHF6; PTPN11; RPS6KA2; RUNX1; SETBP1; SF3B1; SH2B3; SRSF2; STAG2; STK11; SUZ12; TERC; TERT; TET1; TET2; TET3; TP53; U2AF1; ZRSR2* | Full exon region coverage for each gene. Overall *TET2* coverage was low in some cases (<12X) and therefore unable to interpret. | >500X |
| Genoptix Myeloid Molecular Profile | Moffitt Cancer Center | *ASXL1; CBL; DNMT3A; ETV6; EZH2; IDH1; IDH2; JAK2; KIT: MPL;NPM1; NRAS; PHF6; RUNX1; SETBP1; SF3B1; SRSF2; TET2; TP53; U2AF1; ZRSR2* | Gene: Exon tested  *ASXL1: 12-13*  *CBL: 7-9*  *DNMT3A:7-22*  *ETV6: 1-8*  *EZH2:1-19*  *IDH1:2*  *IDH2:4*  *JAK2:12-14 KIT: 8-11, 13, 17-18*  *MPL:10-11*  *NPM1:10-11*  *NRAS:1-3*  *PHF6:1-9*  *RUNX1:3-8*  *SETBP1:3*  *SF3B1:13-16*  *SRSF2:1*  *TET2:1-9*  *TP53:1-10*  *U2AF1:2,6*  *ZRSR2:2-5. 7-11* | >500X |
| TruSeq myeloid Gene Set | Moffitt Cancer Center | *ABL1, ASXL1, CBL, CEBPA, CSF3R, CUX1, DNMT3A, ETV6, EZH2, FLT3, IDH1, IDH2, JAK2, KIT,*  *KMT2A, KRAS, MPL, MYD88, NPM1, NRAS, PHF6, RUNX1, SETBP1, SF3B1, SRSF2, TET2, TP53, U2AF1, WT1 and*  *ZRSR2* | Gene: Exon tested  *ABL: 4-6*  *ASXL1: 12*  *CBL: 8,9*  *CEBPA: full*  *CSF3R: 14-17 CUX1: full*  *DNMT3:full*  *ETV6: full*  *EZH2:full*  *FLT3: 14, 15, 20*  *IDH1:4*  *IDH2:4*  *JAK2:12,14 KIT: 2, 8-11, 13, 17*  *KMT2A: full*  *KRAS: 2,3*  *MPL:10*  *MYD88: 3-5*  *NPM1:10-11*  *NRAS:1-3*  *PHF6: full*  *RUNX1: full*  *SETBP1: 4(partial)*  *SF3B1:13-16*  *SRSF2:1*  *TET2:3-11*  *TP53:2-11*  *U2AF1:2,6*  *WT1: 7,9*  *ZRSR2: full* | >5000x |
